# Effect of an interdisciplinary inpatient program for patients with complex regional pain syndrome in reducing disease activity—a single-center prospective cohort study

**DOI:** 10.1093/pm/pnae021

**Published:** 2024-03-26

**Authors:** Stephanie Schneider, Maria M Wertli, Anna Henzi, Monika Hebeisen, Florian Brunner

**Affiliations:** Department of Physical Medicine and Rheumatology, Balgrist University Hospital, 8008 Zurich, Switzerland; Department of Internal Medicine, Kantonsspital Baden, 5404 Baden, Switzerland; Department of General Internal Medicine, University Hospital Bern, University of Bern, 3010 Bern, Switzerland; Department of Physical Medicine and Rheumatology, Balgrist University Hospital, 8008 Zurich, Switzerland; Department of Biostatistics, Epidemiology, Biostatistics and Prevention Institute, University of Zurich, 8001 Zurich, Switzerland; Department of Physical Medicine and Rheumatology, Balgrist University Hospital, 8008 Zurich, Switzerland

**Keywords:** complex regional pain syndrome, inpatient treatment, multidisciplinary, interdisciplinary

## Abstract

**Objective:**

The aim of this study was to evaluate the benefit of inpatient treatment in reducing disease activity in patients with complex regional pain syndrome (CRPS) who have exhausted outpatient options. Furthermore, the study sought to identify patient-related outcome variables that predict a reduction in disease activity.

**Methods:**

The primary outcome was disease severity (CRPS Severity Score, range 0-16 points). Secondary outcomes included depression, anxiety, physical function, pain interference, fatigue, sleep disturbance, and the ability to participate in social roles and activities, all of which were assessed using the PROMIS-29. Furthermore, pain catastrophizing, neuropathic pain, quality of life, pain self-efficacy, medication intake, and the patient's global impression of change were examined in accordance with current international agreed recommendations, assessed at discharge, 3-month, and 6-month post-discharge. Mixed-effects models were conducted to identify baseline variables associated with CRPS severity.

**Results:**

Twenty-five patients completed the program (mean age 49.28 [SD 11.23] years, 92% females, mean symptom duration 8.5 [SD 6.5] months). Results showed a significant reduction between baseline and discharge of disease activity (CSS −2.36, *P* < .0001), pain (PROMIS-29 pain −0.88, *P* = .005), and emotional function (PROMIS-29 depression −5.05, *P* < .001; fatigue −4.63, *P* = .002). Moderate evidence for a reduction between baseline and discharge could be observed for pain interference (+2.27, *P* = .05), social participation (PROMIS-29 + 1.93, *P* = .05), anxiety (PROMIS-29 −3.32, *P* = .02) and physical function (PROMIS-29 + 1.3, *P* = .03). On discharge, 92% of patients (23 of 25) reported improvement in their overall condition. In the follow-up period, medication intake could be reduced after 3 (MQS −8.22, *P* = .002) and 6 months (MQS −8.69, *P* = .001), and there was further improvement in social participation after 3 months (PROMIS-29 + 1.72, 0.03) and sleep after 6 months (PROMIS-29 + 2.38, 0.008). In the mixed models, it was demonstrated that patients experiencing less pain at baseline also exhibited lower disease activity.

**Conclusion:**

The results of this study confirm that inpatient interdisciplinary treatment of CRPS patients improves disease activity, pain, physical function, emotional function, and social participation. Most improvements were maintained for up to 6 months after discharge. The majority of patients reported that their overall condition had improved during the study period.

## Introduction

Complex regional pain syndrome (CRPS) is a debilitating and often chronic condition that can result in significant physical, psychological, and financial burdens on individuals and their families.^1^ The condition is characterized by a range of symptoms, including pain, sensory disturbances, and motor dysfunction, and is typically triggered by trauma or surgery to a distal extremity.^2^

While some patients experience benign courses, many patients develop a chronic condition with persisting symptoms that can last beyond one year.^3^^,^^4^ This results in high healthcare costs and substantial absenteeism from work, imposing a significant economic burden on affected individuals and society at large.^5–7^ A recent retrospective study conducted in Switzerland analyzed the direct healthcare costs and work incapacity associated with CRPS. Within the first 5 years after the accident, insurance and treatment costs were 19 and 13 times higher, respectively, compared to accidents without CRPS. Two years after the accident, patients with CRPS lost 20 times more days of work (330, 7 days; mean, SD) than patients without CRPS (16.1, 0.1 days; mean, SD). In two-thirds of all CRPS cases, long-term disability lasting more than 90 days developed. Therefore, there is a clear need for effective and evidence-based treatments for patients with persistent CRPS. However, due to the complexity of the disorder, treating it effectively can be challenging. Current therapy is symptom-based and involves various pharmacological, interventional, physiotherapeutic, occupational, and psychiatric treatments.^1^ Treatment is primarily provided in an outpatient setting, but in cases where outpatient treatment options have been exhausted, inpatient treatment options have been shown to be effective in improving function, activity, and social participation.^8–13^ However, the benefits of inpatient treatment in terms of reducing disease activity have not been investigated. Therefore, the objective of this study was to evaluate the benefit of inpatient treatment in reducing disease activity in patients with CRPS who have exhausted outpatient options, assessed at discharge, 3-month, and 6-month post-discharge. Furthermore, the study sought to identify patient-related outcome variables that predict a reduction in disease activity.

## Methods

### Setting and recruitment of study participants

This study was a prospective, observational, single-center cohort study conducted at the Balgrist University Hospital in Switzerland, a privately funded, non-profit hospital, from September 2018 to December 2021. Participants were consecutively recruited from the outpatient clinic of the Department of Physical Medicine & Rheumatology, with all patients admitted for an inpatient program considered for inclusion. Potential participants were informed about the study prior to their attendance at a pre-admission clinic, where eligibility criteria were applied and written informed consent was obtained. The screening visit with potential participants was performed by FB. Patients who opted not to participate in the study still had the opportunity to attend the inpatient interdisciplinary program.

The participants in this study were assessed at admission, discharge, 3-month and 6-month post-discharge. The evaluations comprised various measures of outcomes, such as patient-reported questionnaires, clinician-reported outcomes, and a clinical assessment of presenting signs of CRPS.

The study was approved by the Ethics Committee of Zurich (application BASEC no. 2018-00601) and was reported in line with the STROBE reporting guidelines.^14^

### Inclusion criteria

The inclusion criteria involved adults (age ≥ 18 years) who met the Budapest criteria ^15^ for CRPS in the hand or foot and had a history of unsuccessful pharmacological, physiotherapy, or occupational therapy interventions for at least 6 months.

### Exclusion criteria

Subjects with unconfirmed diagnosis, absence of consent, who lacked proficiency in the German language, and individuals with severe mental health disorders (eg, major depression and schizophrenia) were excluded from the study.

### Description of the treatment program

The treatment program for the patient cohort was partially standardized to allow for individual preferences. The patients were hospitalized for two to three weeks and received interdisciplinary treatment involving various disciplines. A multidisciplinary assessment was conducted upon admission by a physician specializing in rehabilitation medicine, a physiotherapist, an occupational therapist, and a psychologist. Based on the results of the entrance examinations, an individual treatment program was created, which included individual daily physical therapy (pacing, graded exposure, graded motor imagery, mirror therapy, and pain education), individual occupational therapy (reintegration into daily activities, working out weekly/daily structures, and desensitization techniques), group pool therapy and group relaxation training, twice-weekly individual psychological treatment (coping strategies, improving awareness of the affected limb, and medical hypnosis), medical management (coordination of the interdisciplinary setting, continuous review of progress, and adjustment of therapy as needed), and nursing (comprehensive patient care, monitoring vital signs, and medication administration). Daily medical rounds were conducted to review the progress of each patient and drug treatment, which was adjusted as needed, and a grand medical round was held once a week with the responsible physician, nurse, physiotherapist, and social services.

### Outcome measures

The set of CRPS-related parameters is based on the current recommendations of an international research consortium, which considers various aspects of the disease (Core Outcome Measures for complex regional PAin syndrome Clinical Trials [COMPACT]).^16^

#### Primary outcome measure

The primary outcome measure was disease activity assessed using the CRPS Severity Score (CSS).^17^ The score is based on symptoms and findings of the Budapest criteria (16 items, 0 = not present or 1 = present, total of 16 possible points). Higher scores indicate a greater severity of CRPS. A change of 5 or more CSS scale points reflects a clinically significant change.^18^ The CSS has been shown to possess both responsiveness and validity as an effective measure for assessing the severity of CRPS.^18^

#### Assessment of secondary outcomes and further variables

##### PROMIS-29

The Patient Reported Outcomes Measurement Information System 29-item Health Profile (PROMIS-29) is designed to be used with the general population and with individuals living with chronic conditions, and it assesses various aspects of health status, including depression, anxiety, physical function, pain interference, fatigue, sleep disturbance, and ability to participate in social roles and activities.^19^ The measure consists of 28 items from seven domains and a single item on pain intensity. Each item has five response options, except for the pain intensity item which has eleven response options and is measured on a visual analog scale (VAS). The scores generated by PROMIS measures are interpreted based on T-scores with a mean of 50 and a standard deviation of 10 in a reference population.^20^ The Scale ranges from 20 to 80. Depending on the item, a higher score indicates either a positive or negative outcome. Consequently, a higher score on the depression, anxiety, fatigue, sleep, or pain interference item is linked to increased symptoms, whereas a high score on the physical function and social participation item is associated with improved function. In our cohort study, we utilized the existing recommendation from healthmeasures.com to evaluate severity levels, eg, within normal limits, mild (55-60 for pain interference, depression, anxiety, fatigue, and sleep, 45-40 for social participation and activities and physical function), moderate (60-70; 40-30), severe (>70; <30)) based on T-scores across various domains. The clinical validity of the PROMIS questionnaire has been demonstrated across various chronic conditions, such as back pain.^21^

##### Pain Catastrophizing Scale

The Pain Catastrophizing Scale (PCS) is a self-report measure designed to assess catastrophic thinking related to pain among adults.^22^ People are asked to indicate the degree to which they have certain thoughts and feelings related to pain using a 0 (not at all) to 4 scale (all the time). The total score is calculated, ranging from 0 to 52, with higher scores indicating more pain catastrophizing. Scores <30 indicate a not problematic thinking and ≥30 represent problematic levels of catastrophic thinking.^22^ The PCS has been found to have good validity, accurately measuring pain catastrophizing in diverse populations.^22^

##### Short-form McGill Pain Questionnaire-2

The Short-form McGill Pain Questionnaire-2 (SF-MPQ-2) captures neuropathic pain qualities using six neuropathic items.^23^ Each item is rated based on a 0-10 scale with higher scores indicating more neuropathic pain (range 0-60). Based on the results of a Rasch analysis, the Neuropathic Qualities subscale of the SF-MPQ-2 may be used as a standalone measure for neuropathic features in CRPS.^24^

##### EuroQoL 5-dimension 5-level instrument

The EuroQoL 5-dimension 5-level instrument (EQ-5D-5L) is a generic health-related quality-of-life measure that consists of two parts: The descriptive system and the Visual Analogue Scale (EQ-VAS).^25^ The descriptive system consists of five dimensions (mobility, self-care, usual activities, pain/discomfort, and anxiety/depression), each with five response levels, and a total of 3125 health states are defined. Health states are converted into a single index “utility” score using a scoring algorithm. The EQ-VAS provides a single global rating of self-perceived health and is scored on a 0 to 100 mm scale representing “the worst …” and “the best health you can imagine,” respectively. The EQ-5D-5L has been translated and validated in multiple languages and cultural settings, making it suitable for international comparisons of health-related quality of life.^26^

##### Pain Self-efficacy Questionnaire

The Pain Self-efficacy Questionnaire (PSEQ) assesses the confidence people with ongoing pain have in performing activities while in pain.^27^ The questionnaire includes 10 items, and each item is rated on a 0 to 6 scale, with higher scores indicating more confidence (range 0-60). An investigation specifically related to CRPS is lacking; however, a systematic review identified good validity, reliability, and responsiveness for patients with musculoskeletal disorders.^28^

##### Medication Quantification Scale

The Medication Quantification Scale (MQS) is a questionnaire designed for individuals with chronic pain.^29^ The MQS for a drug class is calculated by multiplying the dosage level by the detriment weight, which considers side effects, toxicity, drug interactions, and long-term consequences of the drug. Members of the American Pain Society ranked 22 drug classes on a seven-point scale ranging from no detriment (0) to extreme detriment (6) to determine the detriment weight. Adding all MQS values for individual drug classes provides an overall value for the CRPS medication regimen. The MQS is considered a suitable tool for the investigation of prescription practices and geographic trends in medical pain management in CRPS patients. However, a conclusive statement regarding the relationship between pain intensity and pain medication use cannot be made.^30^

##### Patient global impression of change

The patient global impression of change (PGIC) is a self-reported measure that assesses a patient's overall perception of their treatment response or disease progression over time.^31^ It typically asks patients to rate their status on a 7-point scale ranging from “very much improved” to “very much worse” or a similar rating scale. The PGIC is often used as a complementary measure to other more specific outcome measures and provides valuable information on how patients perceive their health status or treatment outcomes. Global Rating of Change Scales have demonstrated strong construct validity and notable correlations with changes in EQ-5D and Pain Rating Scale. A standardized response mean ranging from 0.5 to 2.7 has been reported.^32^

### Statistical analysis

In the present study, continuous variables were summarized with means and standard deviations (SD), while dichotomous variables were presented as percentages of total. Patient-reported outcomes were treated as continuous variables and subjected to several before-and-after comparisons, including admission and discharge, discharge and 3 months after, and discharge and 6 months after. Paired t-tests were employed for normally distributed data, whereas the Wilcoxon test was used for non-normally distributed data. Due to the limited sample size, continuous data and patient-related outcomes were visually inspected to confirm normal distribution. In addition to reporting the mean differences with a 95% confidence interval (CI), the effect size of the statistical tests was assessed using Cohen's d or r (rank-biserial correlation) for the Wilcoxon test. Effect sizes, as measured by Cohen's d, were interpreted as follows: Values ranging from 0 to 0.2 were deemed negligible, those up to 0.5 were categorized as small, up to 0.8 as moderate, and any value surpassing 0.8 was regarded as indicative of a large effect. In the context of the Wilcoxon test, effect sizes were categorized as follows: Values equal to or exceeding 0.1 were designated as small, those surpassing 0.3 were considered moderate, and an effect size of 0.5 or higher was deemed large. *P* values <.05 were considered statistically significant.

Patient-specific changes were quantified using mixed models, which accounted for potential confounders to minimize systematic errors in inference. Numerical and score endpoints were modeled with linear mixed models, and the number of parameters examined in the model was restricted to accommodate the small sample size. Based on the findings of previous work from Bean et al. we decided to develop a model to investigate the effect of disease activity with adjustments for age, symptom duration, anxiety, and pain intensity at admission.^33^ The effects of the confounding and correction variables were interpreted, and the relevance of potential variation of the effect of correction variables across different time points was assessed with likelihood ratio tests.

All statistical analyses were carried out using R version 3.6.2, while study data was stored and managed using Redcap versions 6.12.1 to 6.14.1 (REDCap, Vanderbilt University, Nashville, TN, United States).

## Results

### Sample description

A sample of 30 patients fulfilled our inclusion criteria and were recruited for this study. One patient opted for premature discharge for personal reasons and therefore, did not complete the program. Four patients opted not to participate in the study due to the extended travel distance. Consequently, a total of 25 datasets were included in the analysis.

### Patient characteristics at admission

The sample comprised primarily of female participants (n = 23, 92%) with a mean age of 49.3 (SD 11.2) years. The upper extremity was more frequently affected (84%) than the lower extremity. Surgical interventions emerged as the most frequent precipitating factor (56%), followed by fractures (28%), bruises (16%), strains (12.7%) and lacerations (4.2%). The average duration of symptoms at admission was 8.5 (SD 6.5) months, with a range spanning from 3 to 32 months. The average length of hospitalization was 16 days (SD 3 days).

### Baseline characteristics

Table 1 presents the baseline characteristics of the enrolled patients. On admission, the disease severity was recorded at 11.4 (SD 2.4) on the CSS scale. The average pain was 5.2 (SD 2.0). The excluded group exhibited similar, non-statistically significant values, with a disease activity score of 11 (SD 3.5, *P* > .05) and PROMIS-29 pain level of 5.2 (SD 2.7, *P* > .05) upon admission.

**Table 1.** Descriptive statistics at admission and discharge.

	Admission	Discharge	
Domain	Mean (SD)	Mean (SD)	Scale^a^
CSS	11.4 (2.4)	9.04 (3.03)	1-16
PROMIS-29			
Pain on VAS (last 7 days)	5.2 (2.0)	4.5 (2.2)	1-10
Physical function	38.6 (5.2)	40.3 (5.1)	80-20
Anxiety	60.8 (6.7)	57.6 (7.8)	20-80
Depression	59.1 (6.9)	54.4 (8.4)	20-80
Fatigue	57.1 (9.9)	52.5 (9.4)	20-80
Sleep disturbance	54.7 (4.9)	53.9 (4.6)	20-80
Pain interference	64.7 (6.9)	62.6 (5.2)	20-80
Social participation	40.1 (6.5)	41.8 (6.1)	80-20
PCS	19.0 (10.41)	17.6 (11.8)	0-52
SF-MPQ-2	34.7 (39.3)	32.5 (41)	0-60
EQ-5D-5L	0.6 (0.2)	0.7 (0.2)	0-1
EQ-5D-5L VAS	58.5 (25.6)	54 (21.9)	0-100
PSEQ	23.8 (14.1)	27.1 (12.9)	0-60
MQS	15.3 (13.6)	13 (12.1)	

a Higher scores are associated with more symptoms, with the exception of EQ-5D-5L, PROMIS-29 physical function and social participation where lower scores are associated with more limitations.

Abbreviations: CSS = CRPS Severity Score; EQ-5D-5L = EuroQoL 5-dimension 5-level questionnaire; EqVAS = Eq Visual Analogue Scale; MQS = Medication Quantification Scale; PCS = Pain Catastrophizing Scale; PROMIS-29 = Patient Reported Outcomes Measurement Information System-29 item; PSEQ = Pain Self-efficacy Questionnaire; SD = standard deviation; SF-MPQ-2 = Short-form McGill Pain Questionnaire 2; VAS = Visual Analogue Scale (pain).

Analysis of the PROMIS-29 questionnaire revealed mild limitations in the domains of depression (59.1, 6.0; mean, SD), fatigue (57.1, 9.9; mean, SD), and social skills (40.1, 6. 5; mean, SD). Conversely, moderate limitations were observed in pain interference (64.7, 6.9; mean, SD), physical function (38.6, 5.22; mean, SD), and anxiety (60.8, 6.7; mean, SD), compared to the reference population.^20^^,^^34^ No sleep disturbances were observed in the subjects.

### Outcomes at discharge, 3-month and 6-month post-discharge

The treatment outcomes are summarized in Table 2.

**Table 2.** Outcomes at discharge, 3-month and 6-month post-discharge.

	Admission to discharge				Discharge to 3 months				Discharge to 6 months			
Domain	Estimate	95% CI	*P*-value	Effect Size (d/r)	Estimate	95% CI	*P*-value	Effect Size (d/r)	Estimate	95% CI	*P*-value	Effect Size (d/r)
CSS^t^	−2.36	−3.35 to −1.37	<.0001***	**0.95^d^**	−0.64	−1.61 to 0.33	0.19	0.26^d^	−0.56	−1.72 to 0.6	0.33	0.19^d^
PROMIS-29												
Pain on VAS^t^	−0.88	−1.46 to −0.29	.0053**	0.61^d^	0.39	−0.09 to 0.87	.11	0.34^d^	0.48	−0.33 to 1.29	.24	0.25^d^
Physical function^w^	1.30	0.20-3.05	.03*	0.45^r^	−0.04	−1.67 to 1.59	.96	0.02^r^	0.50	−1.31 to 2.32	.57	0.07^r^
Anxiety^t^	−3.32	−6.07 to −0.57	.02*	0.5^d^	−1.23	−4.64 to 2.17	.46	0.15^d^	1.20	−2.80 to 5.19	.54	0.13^d^
Depression^w^	−5.05	−7.60 to −2.40	<.001***	0.69^r^	1.42	−1.21 to 4.04	.28	0.3^r^	2.50	−1.52 to 6.52	.21	0.23^r^
Fatigue^t^	−4.63	−7.34 to −1.93	.0018**	0.71^d^	0.62	−3.19 to 4.43	.74	0.07^d^	3.09	−1.96 to 8.14	.22	0.26^d^
Sleep^t^	−1.06	−2.91 to 0.79	.25	0.24^d^	0.25	−1.90 to 2.40	.81	0.05^d^	2.38	0.85-3.95	.0084**	0.5^d^
Pain interference^t^	−2.27	−4.56 to 0.03	.05*	0.41^d^	0.36	−1.22 to 1.94	.64	0.09^d^	0.16	−1.94 to 2.25	.88	0.03^d^
Social participation^t^	1.93	−0.02 to 3.89	.05*	0.41^d^	1.72	0.20 to 3.24	**.03** *	0.46^d^	1.75	−0.70 to 3.95	.19	0.32^d^
PCS^t^	−1.36	−3.99 to 1.27	.30	0.21^d^	−0.48	−4.15 to 3.19	.79	0.05^d^	−2.04	−5.73 to 1.65	.26	0.23^d^
SF-MPQ-2^t^	−2.20	−5.69 to 1.29	.21	0.25^d^	0.36	−4.37 to 5.09	.88	0.03^d^	4.08	−6.08 to 14.25	.41	0.16^d^
EQ-5D-5L^t^	0.06	0-0.12	.04*	0.41^d^	0.03	−0.04 to 0.10	.44	0.15^d^	−0.03	−0.13 to 0.07	.49	0.14^d^
EqVAS^t^	−4.56	−12.25 to 3.13	.23	0.24^d^	−3.60	−13.23 to 6.03	.45	0.15^d^	−0.29	−11.53 to 10.95	.96	0.01^d^
PSEQ^t^	3.28	−0.67 to 7.23	.1	0.33^d^	0.44	−2.26 to 3.14	.74	0.07^d^	−1.5	−5.5 to 2.50	.35	0.17^d^
MQS^w^	−1.75	−4 to 3.90	.4	0.18^r^	−8.22	−13.23 to −3.21	.0024**	0.56^r^	−8.69	−13.51 to −3.87	.0011**	0.62^r^

The difference (t for t-test or w for Wilcoxon test) with 95% confidence interval (CI), the *P* value and the effect size (d for Cohen's D or r for Wilcoxon test) for all three comparisons between admission and discharge, between discharge and the 3-month control, and between discharge and the 6-month control are shown for all domains. Bold indicates statistically significant.

* *P* value < .05;

** *P* value < .01;

*** *P* value < .001.

Abbreviations: CSS = CRPS Severity Score; EQ-5D-5L = EuroQoL 5-dimension 5-level Questionnaire; EqVAS = Eq Visual Analogue Scale; MQS = Medication Quantification Scale; PCS = Pain Catastrophizing Scale; PROMIS-29 = Patient Reported Outcomes Measurement Information System-29 item; PSEQ = Pain Self-efficacy Questionnaire; SF-MPQ-2 = Short-form McGill Pain Questionnaire 2.

### Primary outcome

**Figure 1.**
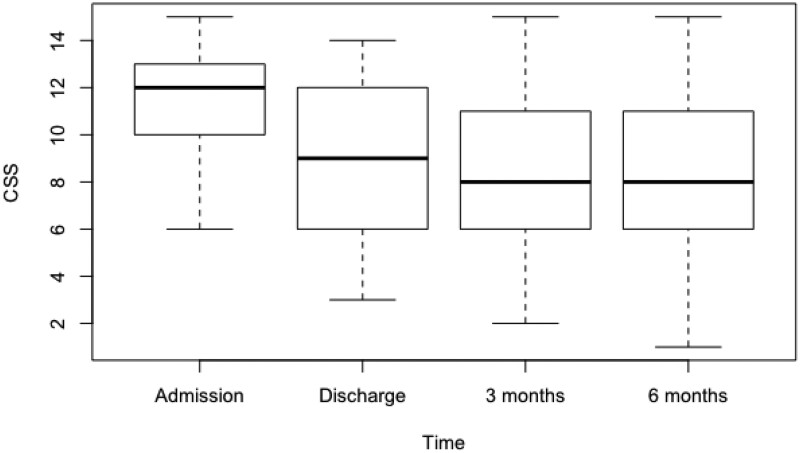
CRPS severity score boxplot.

Upon discharge, disease activity decreased significantly compared to baseline (CSS −2.36 [95% CI, −3.35 to −1.37], *P* < .0001, large effect size d = 0.95). No statistically significant changes were observed at 3- and 6-month follow-ups. The visual trajectory is illustrated in Figure 1.

### Secondary outcomes

On discharge, some evidence for an improvement was observed in the following domains of the PROMIS-29: Depression (−5.05 [95% CI, −7.6 to −2.4], *P* < .001, large effect size r = 0.69), anxiety (−3.32 [95% CI, −6.07 to −0.57], *P* = .02, small effect size d = 0.5), physical function (1.3 [95% CI, 0.2-3.05], *P* = .03, moderate effect size r = 0.45), Pain interference (−2.27 [95% CI, −4.56 to 0.03], *P* = .05, small effect size d = 0.61), pain intensity (−0.88 [95% CI, −1.46 to −0.29], *P* = .0053, moderate effect size d = 0.41), fatigue (−4.63 [95% CI, −7.34 to −1.93], *P* = .0018, moderate effect size d = 0.71,) and social participation (1.93 [95% CI, −0.02 to 3.89], *P* = .05, small effect size d = 0.41). These improvements persisted throughout the subsequent 3- and 6-month follow-up periods.

There were no statistically significant changes observed in pain catastrophizing, pain self-efficacy, or neuropathic pain.

We observed an improved quality of life on the EQ-5D-5L (0.06 [95% CI, 0-0.12], *P* = .04, small effect size d = 0.4) at discharge, which was sustained at 3- and 6-month follow-ups.

There was no evidence of alteration in the quantity of medication administered during the inpatient stay. However, during the period between discharge and 3 months after, and also between discharge and 6 months after, there was a statistically significant reduction in medication usage, indicated by MQS values of −8.22 (95% CI, −13.23 to −3.21, *P* = .0024, large effect size r = 0.56) and −8.69 (95% CI, −13.51to 3.87, *P* = .0011, large effect size, r = 0.62), respectively.

### Patient global impression of change (PGIC)

**Figure 2.**
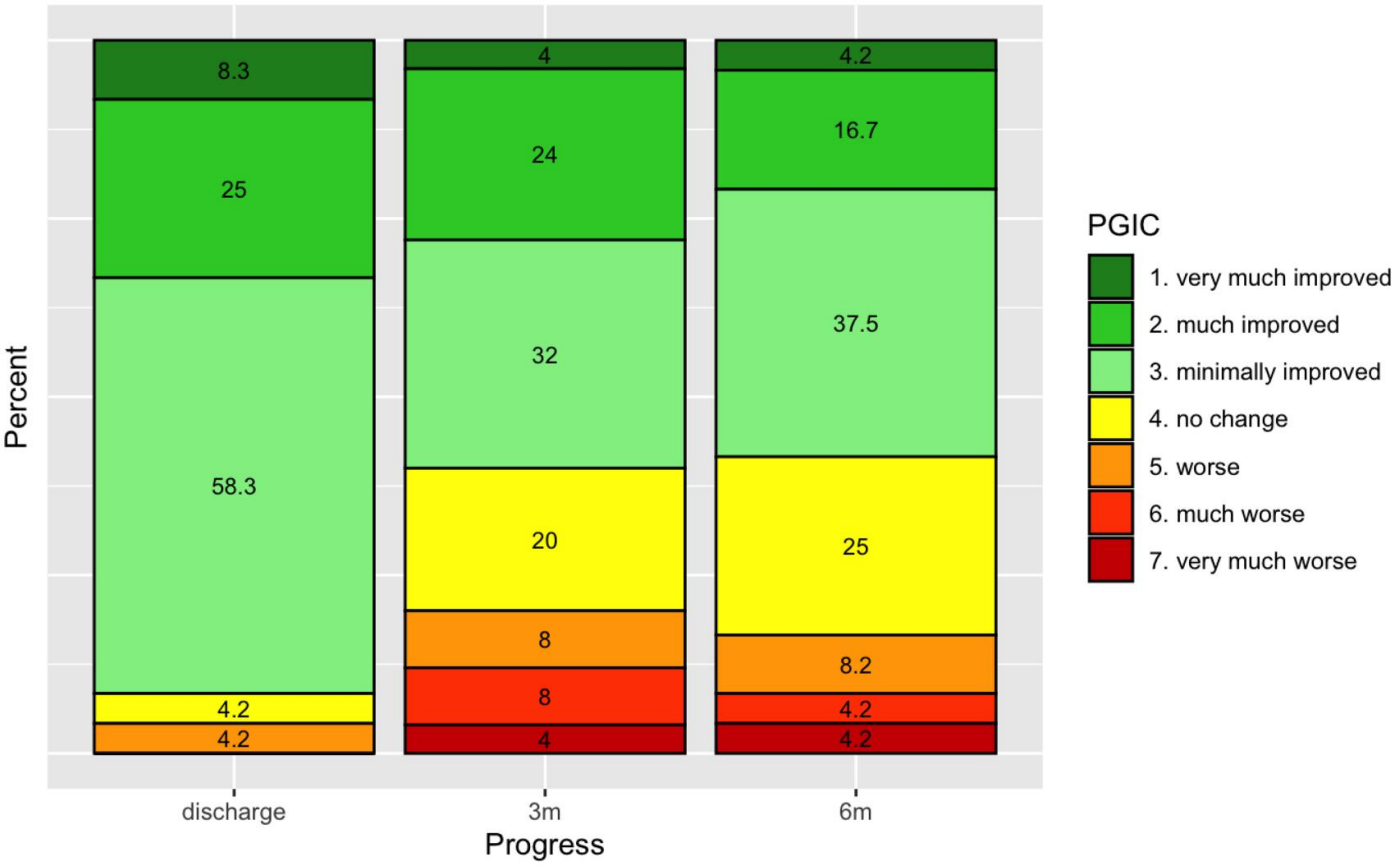
Patients global impression of change.

On discharge 23 of 25 patients (92%) reported improvement in their overall condition. Three-month and 6-month post-discharge 15 of 25 (60%) respectively 14 of 24 (58%) of the patients still reported further improvement in their overall condition when compared to discharge. The patient's global impression of change is depicted in Figure 2.

### Course of CRPS symptoms adjusted for potential confounders

In the linear mixed effect model (Table 3) similar changes of CSS were calculated as in the unadjusted comparisons (admission to discharge −2.22 [95% CI, −3.37 to −1.06], *P* < .001). Consequently, CSS effects apply, regardless of confounding and adjustment factors. Patients who experienced greater pain at admission (+0.76 CSS per +1 pain [95% CI, 0.25-1.28], *P* = .0038) showed increased disease activity. No association was detected between CSS and symptom duration, anxiety at admission, and age. Similarly, no variation in the association between CSS and pain or anxiety was observed at different time points.

**Table 3.** Multivariate analysis of symptom severity after inpatient treatment up until 6 months follow-up.

	CRPS Severity Score		
	Estimate^a^	95% CI	*P*-value
Age	−0.05	−0.15 to 0.04	.3
Symptom duration	0.08	−0.06 to 0.23	.28
Anxiety (PROMIS-29)^b^	−0.15	−0.17 to 0.14	.88
Pain intensity (PROMIS-29)^b^	0.76	0.25-1.28	.0038*
Admission to Discharge^c^	−2.22	−3.37 to −1.06	<.001**
Discharge to 3 months^c^	0.7	−0.46 to 1.85	.24
Discharge to 6 months^c^	0.74	−0.41 to 1.89	.21

* *P* value <.01;

** *P* value <.001.

a Comparison of outcome between admission, 3 months and 6 months compared to discharge with adjustment for age, symptom duration, anxiety, and pain.

b Pain intensity and anxiety assessed at Admission (Baseline).

c Course of disease activity at different times after adjustment of the above-mentioned variables.

## Discussion

The aim of this study was to evaluate the benefit of inpatient treatment in reducing disease activity in patients with CRPS who have exhausted outpatient options, assessed at discharge, and at 3-month and 6-month post-discharge. The results of our study revealed a reduction in disease activity from admission to discharge, a trend that persisted during the 3- and 6-month follow-up assessments. Linear mixed models confirmed the findings of the unadjusted comparisons. Additionally, a relationship between pain intensity upon admission and disease activity was established.

### Results in the light of the existing literature

Due to the complexity and the multifaceted nature of CRPS, the therapy remains a challenge and no causal therapy is available.^3^^,^^8–11^^,^^35^ In the past, several studies showed that an interdisciplinary inpatient treatment is effective in improving CRPS symptoms.^9^^,^^10^^,^^12^ The main outcome measure of these studies was a reduction of pain on the numeric rating scale. Additional parameters included physical function measured by range of motion and grip strength of the affected hand,^9^ body perception disturbance and emotional function ^10^ and perception and management of pain.^12^ Multidisciplinary outpatient treatment programs also focused on pain reduction,^8^^,^^11^ physical function ^8^^,^^11^^,^^13^ and psychological factors such as depression, anxiety and pain acceptance.^8^^,^^11^^,^^13^ To harmonize the outcome measures of clinical studies, an international committee has developed and validated the CRPS severity score (CSS).^36^^,^^37^ Elomaa et al. have used a similar CRPS symptom count for their inpatient study before CSS was developed, and showed that motor, trophic and sensory symptoms improved. So far, only one study used the CSS as main outcome parameter,^3^ however this study assessed the disease course of outpatients with no specific or predefined treatment regime.

The present study is the first to show that inpatient treatment is able to reduce disease activity as assessed by CSS. We observed a significant reduction in disease activity from admission to discharge, averaging −2.36 points on the CSS. In Bean et al. 2016, CSS in patients with recent diagnosis of CRPS (< 12 weeks) showed the greatest reductions in disease activity during the first 6 months of outpatient treatment. In this study, the mean of the baseline CSS score was 12.6 (SD 2.0) and thus slightly higher than in our work (mean 11.4, SD 2.4). The patients in our study were admitted to inpatient treatment only after a mean symptom duration of 8.5 months when outpatient treatment options were exhausted. The fact that we observed a reduction in disease activity during hospitalization suggests that inpatient treatment is effective in patients with stagnant activity in longstanding disease. It is important to mention that the change in CSS observed in our study does not meet the threshold for clinical significance of > 4.9 points on CSS as suggested by Harden et al. 2017 ^18^. This threshold was calculated based on the comparison of disease course in patients with more recent diagnosis of CRPS and newly initiated treatment to patients with longer disease duration on stable treatment regimes, and should reflect the smallest real difference under the assumption that the stable treatment group showed no clinically relevant change in disease activity. However, it should be noted that this threshold has not been externally validated. Since the patients in our study reported a significant and lasting improvement in physical function, emotional wellbeing and participation in activities of daily life, we suspect that a reduction in CSS < 4.9 points might correspond to a clinically relevant improvement. Alternatively, the CSS score might be more appropriate to detect longer-term changes in disease activity as assessed by Harden et al. 2017 ^18^ and Bean et al. 2016 ^3^ (3 and 6 months) than the benefit of shorter interventions such as inpatient treatment programs.

While the duration of therapy had no influence on pain reduction in the study by Kotsougiani-Fischer et al., Raqué et al. were able to demonstrate that patients receiving therapy early experienced better pain reduction than patients with longstanding disease (symptom duration < 12 months and > 24 months respectively). In our study, we found no correlation of change in CSS with symptom duration. However, our study included only very few patients with symptom duration of > 24 months.

We observed improvements in psychological functioning, particularly in depressive symptoms, anxiety, and fatigue. This is in line with some previous studies,^3^^,^^11^ while two studies reported no significant reductions in anxiety or emotional wellbeing.^8^^,^^13^ In a prospective study by Bean et al. 2015, patients with less pain-related and general anxiety had less disability and pain.^33^ De Jong et al. also found an association between pain-related anxiety and function of the affected limb.^38^ It has been suggested that anxiety is linked to an increased inflammatory state ^39^ and sympathetic activity, which are both implicated in the development of CRPS.^40^ In contrast to these results, we detected no association between anxiety and disease activity. However, we did not assess pain-related anxiety specifically.

Similar to McCormick et al. 2015, we found no significant decrease in medication use during the treatment program. Two factors contribute to the constancy of medication use between the initial assessment and discharge. Firstly, some patients experienced severe pain that persisted despite exhausting their current medication dosage, necessitating a switch to alternative medication classes during their inpatient stay. Secondly, some patients had previously avoided using the affected limb due to pain and required analgesics to enable their active participation in the intensive physiotherapy and occupational therapy program. Like McCormick et al., we found a reduction in medication use in at the follow-up examinations, indicating the success of the therapy.

### Strength and limitations

Our study has several strengths. In addition to individual complaints occurring in the context of CRPS, such as pain or functional limitations, we documented the overall health of the patients by means of different domains described in the methods section according to COMPACT.^16^ This allows to more adequately capture the multi-layered nature of the disease. Furthermore, by reducing the heterogeneity of the documented parameters, a comparison with future studies should be simplified, and thereby lead to better evidence for the investigation of this rare disease. To the best of our knowledge, this study is the first to investigate the long-term effectiveness of a multi-modal inpatient therapy with consideration of the above-mentioned parameters.

Since the data for this study were collected prospectively and the patients were included according to established diagnostic criteria, sampling biases, such as misdiagnosed patients, are reduced.

However, it's important to acknowledge some limitations. The severity thresholds used for the PROMIS-29 questionnaire have been established for rheumatoid conditions and cancer. Therefore, despite similarities with CRPS patients, these values should be interpreted with caution within the context of our cohort study. Secondly, the lack of randomization and control group is considered a weakness of this study. Furthermore, due to the small sample size the generalizability of the results is limited. Finally, our study does not allow specific assessments of individual treatment modalities, since treatments are considered in combination in the form of an interdisciplinary therapy.

### Implication for research

In order to accurately assess the effectiveness of inpatient treatment for CRPS patients and determine the appropriate patient groups for therapy, randomized controlled trials with a large sample size are necessary. While this study contributes to the existing evidence supporting clinical, evidence-based inpatient therapy for select cases, it cannot provide definitive recommendations for an optimal combination of treatment options that cater to all CRPS patients. Further research is required to enhance our understanding of the disease's underlying mechanisms and to investigate the efficacy and dosages of individual modalities in relation to CRPS.

## Conclusions

Inpatient interdisciplinary treatment for CRPS patients who have exhausted outpatient options shows promising improvements in disease activity, pain, physical function, depression, anxiety, fatigue, and social participation. However, further studies, preferably controlled, multicenter, or randomized controlled trials, are needed to validate the effectiveness of this approach and investigate potential advantages of inpatient therapy or day hospital programs over outpatient care. These studies would contribute to a better understanding of optimal treatment strategies for CRPS patients.
